# Associations of Circadian and Metabolic Syndromes with Cardiovascular Diseases and Diabetes in Qatar: A Cross-Sectional Study

**DOI:** 10.2147/DMSO.S503143

**Published:** 2025-09-04

**Authors:** Shahd Hamran, Omar Altrmanini, Mhd Osama Rahhal, Raneem Alsheikh, Iman Amro, Giridhara Rathnaiah Babu, Muhammad Naseem Khan, Habib Hasan Farooqui, Tawanda Chivese, Salma M Khaled

**Affiliations:** 1 College of Medicine, Qatar University, Doha, Qatar; 2 Social and Economic Survey Research Institute, Qatar University, Doha, Qatar; 3 Department of Population Medicine, College of Medicine, Qatar University, Doha, Qatar

**Keywords:** metabolic syndrome, circadian syndrome, diabetes mellitus, cardiovascular diseases, Qatar

## Abstract

**Objective:**

Metabolic syndrome (MetS) increases the risk of cardiovascular diseases (CVDs). A combination of MetS with Circadian rhythm disorder (CRD) may be a stronger risk factor for CVDs than MetS alone. We conducted a nationally representative cross-sectional study to compare the associations of CRD and MetS with type 2 diabetes (T2DM) and CVDs in Qatar.

**Methods:**

Sociodemographic and health information were collected. MetS status was determined based on standard international criteria. CRD was defined based on either i) short sleep duration (≤ 6 hours per night) and MetS or (ii) short sleep duration, self-reported depression (or PHQ-2 score ≥ 3) and two components of MetS.

**Results:**

A total of 2523 respondents completed the interview, with a mean age of 37.1 years (SD = 11.2), women (n=637, 27.3%), and Qataris (n=754, 32.3%). The overall prevalence of MetS and CRD was 6.8% (95% CI: 5.4–8.5) and 2.4% (95% CI: 1.7–3.4), respectively; MetS was more prevalent in men (7.0%) compared to women (5.8%); the opposite was true for CRD (women 4.4% vs men 2.4%). Older age was a predictor of both MetS and CRD. Compared to Qataris, blue-collar expatriates had lower odds of MetS (OR = 0.32, 95% CI 0.23–0.58) and CRD (OR = 0.46, 95% CI: 0.20–1.05). Current married status was positively associated with MetS, but not CRD. Both MetS (OR=19.08, 95% CI: 10.87–33.50) and CRD (OR=10.32, 95% CI: 4.60–23.17) were strongly associated with T2DM. Similarly, MetS (OR = 5.51, 95% CI: 2.33–13.03) and CRD (OR = 2.01, 95% CI: 0.92–4.42) were associated with CVDs.

**Conclusion:**

MetS showed potentially stronger associations than CRD with T2DM and CVDs in Qatar. Further research is needed into the predictive utility of CRD compared with MetS for these outcomes in different populations including the Middle East.

## Introduction

The circadian system is the body’s internal clock that regulates sleep-wake cycles, hormone secretion, and metabolism. Several lifestyle behaviors have been attributed to the disruption of this system including shift work, jet lag, irregular sleep patterns, and exposure to artificial lights at night.^1^ The interplay between circadian disruptions and metabolic dysregulation highlights the complicated relationship between sleep patterns (quantity and quality of sleep) and metabolic balance, specifically glucose metabolism. Evidence from primary studies shows a positive relationship between circadian disturbances and glucose intolerance, obesity, and insulin resistance leading to the development of type 2 diabetes (T2DM).^2–6^ In Qatar’s population, preliminary evidence supported a positive association between short sleep duration and poor self-rated health, obesity, and chronic illness independent of age, gender, or social class.^7^

Metabolic syndrome (MetS) – a marker of metabolic dysfunction – includes obesity, glucose intolerance, dyslipidemia and hypertension, and insulin resistance is strongly associated with an elevated risk of T2DM, cardiovascular diseases (CVDs), mood disturbances, and mortality.^8^ The metabolic abnormalities in MetS such as insulin resistance and glucose intolerance further exacerbate hyperglycemia and T2DM complications, and once T2DM develops, it often worsens other components of MetS, such as lipid profile, creating a vicious cycle that amplifies CVDs risk.^9^ According to the American Heart Association (AHA), MetS is defined by the presence of three or more of the following: fasting blood glucose ≥ 100 mg/dL; waist circumference ≥ 88 cm for women and ≥ 102 cm for men; elevated blood pressure represented by systolic pressure of ≥130 and/or diastolic of ≥85 mm Hg and/or use of antihypertensives; HDL cholesterol < 40 mg/dL for men and < 50 mg/dL for women; and triglycerides ≥ 150 mg/dL. The Joint Interim Statement definition (JIS) definition for MetS is identical to the AHA components, but considers patients on glucose control medication, or LDL lowering or HDL raising medicines as alternative in diagnosis to their corresponding laboratories abnormalities.^10^ Furthermore, due to lifestyle changes, in 2022, the prevalence of MetS worldwide was around 31.4% based on the JIS and the incidence of MetS is increasing particularly in the Middle East region, where prevalence estimates are as high as 34% according to the JIS criteria.^11^ The consequences of MetS are not limited to CVDs, with growing evidence of its association with mood disorders such as depression according to several meta-analyses.^12–14^

In contrast to MetS, Circadian Metabolic Syndrome or Circadian Syndrome (CRD), is a relatively new concept, which has been described in some primary studies.^15–17^ However, no clear definition exists. Given the close relationship between the circadian and metabolic systems, some authors proposed to incorporate depression, metabolic dysfunction-associated steatotic liver disease (MASLD), and sleep-wake cycle disruptions into the definition for this syndrome.^17^

Evidence from the National Health and Nutrition Examination Survey, a study based in the United States, showed high odds of CVD mortality in participants with MetS and CRD. However, the odds of mortality were higher in individuals with CRD than MetS.^15^ There is limited evidence from Middle Eastern populations regarding the association between CRD, MetS, and CVDs.

Our study aimed to estimate the prevalence of CRD and MetS and identify their potential risk factors. Additionally, due to Qatar’s distinctive demographic composition and lifestyle characteristics, including potentially high rates of shift work and widespread use of artificial lighting, it was pertinent to investigate the associations of CRD and MetS with T2DM and CVDs to delineate primary and secondary prevention pathways and guide relevant public health initiatives and policy adjustments.

## Methods

### Study Design

A cross-sectional study was conducted between March and April of 2018. The interview questionnaire was designed to assess health and diabetes mellitus awareness in Qatar among its citizens and expatriates.

A stratified probability-based sample was drawn from a cellphone frame prepared by the Social and Economic Survey Research Institute (SESRI) with the help of the main telecommunication providers in Qatar. The sample was representative of the three main groups within Qatar’s resident population (Qatari nationals, white-collar expatriates (WCE), and blue-collar expatriates (BCE)).^18^ A total of 10,579 phone interviews were attempted, of which 5872 constituted our target population of adults who were 18 years or older and living in Qatar at the time of the study. Up to seven call attempts were made to complete the interview for each participant. After removing “hard refusals” and numbers that were difficult to reach, a total of 2560 interviews were completed giving an overall response rate of 43.6%. After data cleaning (N = 2523) and removing cases for which MetS or CRD status could not be determined (n=191), a final sample of 2332 was retained for the analysis.

### Interview Questionnaire Development

The interview questions were initially developed in English and then translated into Arabic and other languages (Urdu, Hindi, Malayalam, Nepalese, Bengali and Tamil) by professional translators. The translated version was carefully checked by bilingual researchers using forward-backward translation. The interview questionnaire was programmed into the CATI (Computer Assisted Telephone Interview) system using Blaise software. A pre-test was run on a small number of cellphone units, which informed the further refinement of the questionnaire before study fielding.

### Interview Administration

Telephone interviews were conducted by a team of experienced interviewers and supervisors at Social and Economic Survey Research Institute (SESRI). The phone calls were allocated over times of day and days of the week to maximize the chances of contacting respondents. The interviews took on average between 20 and 30 minutes to complete.

### Data Management

After the data collection, all individual interviews were merged and saved in a single Blaise data file. This dataset was cleaned, coded, and weighted to account for complex sample design including sampling selection probability, non-response, and calibration to align estimates with known population characteristics available from the census bureau.^19^

### Main Study Measures and Outcomes

International guideline consider individuals having three or more of the following criteria as having MetS: high cholesterol levels, high triglyceride levels, high waist circumference (> 85cm), prediabetes, and raised blood pressure (DBP ≥ 85mmHg or SBP ≥ 130mmHg).^10^

All variables used to ascertain the abovementioned criteria for MetS and define our main outcomes T2DM and CVDs were defined based on yes responses to a series of questions prefaced by “have you ever been told by a health professional such as a nurse or a doctor that you have any of the following health conditions?”… the list included: “Cardiovascular or heart disease?”, “Diabetes mellitus?”, “Hypertension or high blood pressure?”, “high cholesterol?”, “high triglycerides?”, among other chronic health conditions. For example, if they said yes to any of these questions, then they were considered positive for that criterion of MeTS. The exact wording for survey questions used to define our main exposures and outcomes appear in Appendix A.

Of those who reported having diabetes mellitus, they were further asked if they knew which type of diabetes they had (type 1 or T2DM).

Depression was defined as having at least a total score of 3 out of 6 on the two-item or Patient Health Questionnaire 2 (PHQ-2) in the past two weeks.^20^ The number of hours of sleep on a typical night were used to define average duration of sleep for each participant in the survey.

CRD was defined based on criteria adapted from previous studies [14, 15] of either (i) short sleep duration (≤ 6 hours per night) and MetS or (ii) short sleep duration, depressive symptoms ((PHQ-2) score ≥ 3) and two components of MetS.

Current smoking status was assessed by the following question: “At the present time, do you smoke cigarettes daily, occasionally or not at all?”, for which, responses were grouped into current versus non-current smoker (never and former smoker status).

Other covariates were age, gender, income status, marital status, and education level. Age was recorded as a date of birth, which was subtracted from the interview year (2018) to derive an estimate of actual age in years. The respondent type was used as a proxy for socioeconomic status in our study, reflecting three main working classes in Qatar: Qatari citizens, white-collar or blue-collar expatriates. Respondent type was ascertained by asking a series of questions about their nationality and per month salary ranges in Qatari Riyals (QAR): 1) Qataris (less than 30K, 30K to 50K, 50K to 70K, or 70K QAR or more); 2) white-collar expats (4K to 10K, 10K to 15K, 15K to 25K, 25K QAR or more); and 3) blue-collar expats (Less than 1K, 1K to 1.4K QR, 1.4K to 1.8K QR, and 1.8K to 4K QAR).^18^ For marital status, respondents were categorized into two categories based on their status: 1) married and 2) not married, which included separated, divorced, widowed and never married. Education level was divided into school education (Primary (1–6), Preparatory (7–9), Vocational (After Preparatory, but not Secondary), and Secondary (10–12)), university education (Diploma, Bachelor of Arts (BA), Bachelor of Commerce (BCOM), Bachelor of Science (BSC), Master’s degree, and Doctor of Philosophy (PHD)), never attending school, and other.

### Data Analysis

Our statistical analysis was conducted using Stata software (version 18) (StataCorp LLC, College Station, TX, USA). Descriptive statistics, bivariable comparisons, and multivariable logistic regression were carried out. To account for complex sampling design in our estimates, we applied sampling weights using the survey (svy) commands in Stata. For our descriptive results, we reported frequency in total sample of each variable. For bivariable results, weighted proportions of each of our exposure groups (MetS, CRD, no MetS or CRD) were reported with corresponding 95% confidence intervals (CI) and compared across different levels of sociodemographic and health characteristics. The F-transformed version of the Pearson Chi-squared statistics were used to generate the corresponding p-values for these comparisons. Multivariable logistic regression models for estimating the associations between a set of different risk factors and each of our main exposure variables (MetS, CRD), with sampling weights, were fit to data. We then used this minimum set of confounders to estimate the main effects of each independent variable (MetS, CRD) on CVDs and T2DM in lieu of a causal model rather than fitting predictive models for these outcomes.^21^ The overall model fit for these logistic regression models was assessed using the log-likelihood ratio, the Akaike information criterion (AIC) and Bayesian information criterion (BIC). Model misclassification was assessed using the area under the curve (AUC) method.

### Ethical Considerations

The ethics approval for the study was granted by the Ministry of Health (MOPH-A-QDA-007) and our university’s ethics committee (QU-IRB 882-E 2018). We conducted a minimal-risk phone survey interview, which qualified for a verbal consent process over the phone. Our institutional review board (IRB) reviewed and approved our interview questionnaire and the phone script that addressed key elements of informed consent. Each participant was allocated a unique case number at the beginning of the interview. The data was anonymously stored on a highly secure university server.

## Results

### Characteristics of Participants and the Prevalence of MetS and CRD

As shown in Table 1, the total sample of participants that completed the interview was 2332. The mean age of the participants was 37.1 years (SD = 11.2), 637 (27.3%) were women, and 754 (32.3%) were Qataris.

**Table 1:** Sample Characteristics by Metabolic Syndrome (MetS) and Circadian Syndrome (CRD)

Variable		Frequency in Total Sample (N=2332)	MetS N = 207 n (%)	CRD N = 102 n (%)	No MetS or CRD N = 2023 n (%)	P-value
**Age (Years)**	**< 40**	1422	56 (3.4)	33 (1.4)	1333 (95.2)	<0.001
	**≥ 40**	814	146 (15.1)	65 (5.8)	603 (79.1)	
	**Missing**	96	5 (2.0)	4 (2.4)	87 (95.6)	
**Gender**	**Male**	1695	151 (7.0)	66 (2.4)	1478 (90.6)	0.2954
	**Female**	637	56 (5.8)	36 (4.4)	545 (89.8)	
**Socioeconomic Status**	**Qataris**	754	89 (12.0)	51 (7.0)	614 (81.0)	0.002
	**WCE**	893	86 (8.9)	34 (3.1)	773 (88)	
	**BCE**	685	32 (5.3)	17 (2.1)	636 (92.6)	
**Education Level**	**School Education**	1103	82 (6.4)	45 (2.5)	976 (91.1)	0.0288
	**University**	1167	119 (7.8)	56 (3.8)	992 (88.4)	
	**Never**	49	3 (1.6)	1 (0.4)	45 (98.0)	
	**Other**	8	3 (49.4)	0 (0.0)	5 (50.6)	
	**Missing**	5	0 (0.0)	0 (0.0)	5 (100.0)	
** Marital Status**	**Married**	1629	175 (8.3)	82 (3.2)	1372 (88.5)	0.0013
	**Not Married**	698	32 (1.9)	20 (1.4)	646 (96.7)	
	**Missing**	5	0 (0.0)	0 (0.0)	5 (100)	
**Smoking Status**	**Yes**	745	60 (7.0)	25 (1.2)	660 (91.8)	0.1005
	**No**	1587	147 (6.7)	77 (3.5)	1363 (89.8)	
	**Missing**	0	0 (0.0)	0 (0.0)	0 (0.0)	
**Cardiovascular Diseases (CVDs)**	**Yes**	70	23 (26.5)	12 (14.5)	35 (59.0)	<0.001
	**No**	2260	183 (6.3)	90 (2.5)	1987 (91.2)	
	**Missing**	2	1 (50.0)	0 (0.0)	1 (50.0)	
**Type 2 Diabetes Miletus (T2DM)**	**Yes**	256	103 (40.2)	46 (17.9)	107 (41.8)	<0.001
	**No**	2075	104 (5.0)	56 (2.7)	1915 (92.2)	

**Note**: Weighted percentages are different from raw percentages based on the frequency of observations.

**Abbreviations**. WCE, White-collar Expatriate; BCE, Blue-collar Expatriate.

The overall prevalence of MetS and CRD was 6.8% (95% CI: 5.4–8.5) and 2.4% (95% CI: 1.7–3.4), respectively. MetS and CRD were more prevalent in the population above 40 years of age (15.1% and 5.8%, respectively. P≤0.001). Overall, MetS was more prevalent in men (7.0%) compared to women (5.8%); CRD showed a different pattern with higher prevalence in women (4.4%) than men (2.4%), however these differences were not statistically significant (p=0.2954). MetS was more common among married (8.3%) participants than CRD (3.2%) with p-value of 0.0013. In addition, being a smoker was more prevalent among those with MetS (7.0%) than CRD (1.2%) (p=0.1005) (Table 1). CVDs were more prevalent among those with MetS (26.5%) than those with CRD (14.5%) (Table 1).

### Risk Factors for MetS and CRD

Unitary increases in age were associated with both MetS and CRD with OR of 1.06 in both being statistically significant (p<0.001) (Table 2). Socioeconomic status was negatively associated with both MetS and CRD. Compared to Qataris, WCE had lower odds of MetS (OR = 0.56, 95% CI 0.40–0.79) and CRD (OR = 0.57, 95% CI: 0.32–0.99), respectively. Similarly, BCE had lower odds of MetS (OR = 0.32, 95% CI 0.23–0.58, p<0.001). However, association between BCE and CRD (OR = 0.46, 95% CI: 0.20–1.05) were not statistically significant (p=0.066). As shown in Table 2, there was a statistically significant association between currently married compared unmarried participants and MetS (OR = 2.39, 95% CI: 1.56–3.66, p<0.001), but not with CRD (OR=1.44, 95% CI: 0.74–2.79, p= 0.270).

**Table 2:** Risk Factors for Metabolic Syndrome (MetS) and Circadian Syndrome (CRD) From Multivariable Regression Models

		MetS			CRD		
		OR	95% CI	p-value	OR	95% CI	p-value
**Age** (Years)		1.06	(1.04 −1.09)	<0.001	1.06	(1.03 −1.08)	<0.001
**Gender** (Ref. Male)	**Female**	0.70	(0.46 −1.07)	0.108	1.44	(0.71–2.91)	0.304
**Socioeconomic Status** (Ref. Qataris)	**WCE**	0.56	(0.40 −0.79)	<0.001	0.57	(0.32–0.99)	0.047
	**BCE**	0.32	(0.23 −0.58)	<0.001	0.46	(0.20–1.05)	0.066
**Smoking Status** (Ref. Non-smoker)	**Yes**	1.02	(0.60 −1.75)	0.917	0.50	(0.26–0.96)	0.038
**Marital Status** (Ref. Not Married)	**Married**	2.39	(1.56 −3.66)	<0.001	1.44	(0.74–2.79)	0.270
**Education Level**	**School Education**	3.48	(0.94–12.80)	0.061	2.97	(0.37–23.31)	0.299
	**University Education**	3.68	(2.76–147.03)	0.003	3.03	(0.38–23.83)	0.292

**Abbreviations**: OR, Odds Ratio; CI, Confidence interval; Ref, Reference. WCE, White-collar Expatriate; BCE, Blue-collar Expatriate.

### MetS and CRD as Risk Factors for T2DM and CVDs

As shown in Table 3, both MetS (OR = 5.51, 95%: CI 2.33–13.03) and CRD (OR = 2.01, 95% CI: 0.92–4.42) had increased odds of CVDs, however, CRD results did not demonstrate statistical significance (p=0.080). Additionally, MetS showed had higher odds of T2DM (OR = 19.08, 95% CI: 10.87–33.50) compared to CRD (OR = 10.32, 95% CI: 4.60–23.2).

**Table 3:** Associations Between Cardiovascular Diseases (CVDs) and Type 2 Diabetes (T2DM) and Both Metabolic Syndrome (MetS) and Circadian Syndrome (CRD)

Variables	T2DM			CVDs		
	Odds Ratio	95% CI	p- value	Odds Ratio	95% CI	p-value
**MetS**	19.08	(10.87–33.50)	<0.001	5.51	(2.33 −13.03)	<0.001
**CRD**	10.32	(4.6–23.17)	<0.001	2.01	(0.92–4.42)	0.080

**Note**: Both models controlled for age, gender, Socioeconomic Status, Smoking Status, and Marital Status.

**Abbreviations**: CI, Confidence interval; CRD, Circadian syndrome; MetS, Metabolic syndrome.

**Figure 1:**
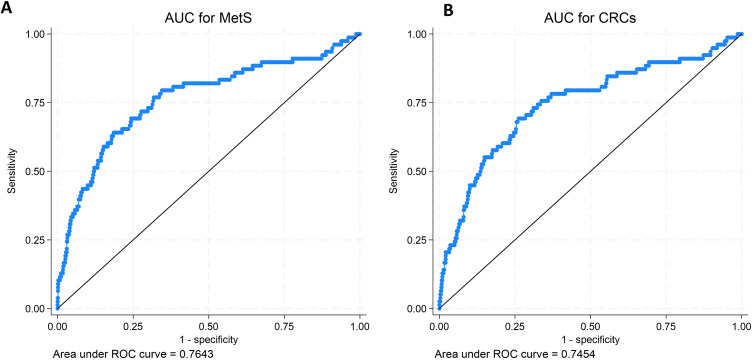
Receiver Operator Curves (ROCs) for models with Metabolic Syndrome (MetS) and Circadian Syndrome (CRD) Regressed on Cardiovascular Diseases (CVDs). The figure on the left (**A**) shows the area under the curve (AUC) for the receiver operating curve (ROC) of the multivariable model with MetS regressed on CVDs. The figure on the right (**B**) shows the AUC of the ROC for the multivariable model of CRD regressed on CVDs.

The AUC values (MetS = 0.764 vs CRD = 0.745) for models shown in Table 3 and their corresponding ROC curves indicated that models for MetS had marginally better predictive capacity for CVDs than CRD models (Figure 1). For MetS, AIC and BIC values were 614.97 and 661.17, respectively. For CRD, the AIC was 632.50 and BIC was 678.70, respectively.

**Figure 2:**
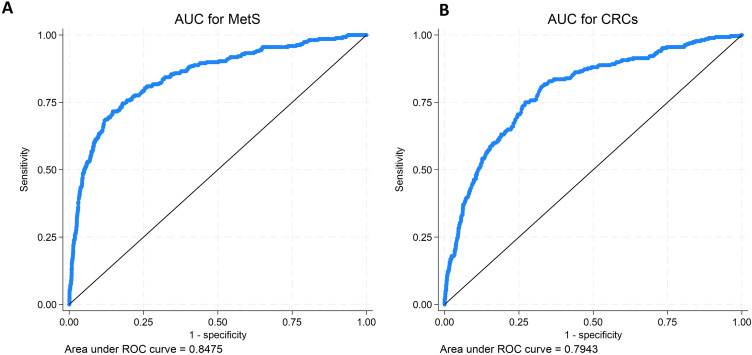
Receiver Operator Curves (ROC) for Models of Metabolic Syndrome and Circadian Syndrome Regressed on Type 2 Diabetes (T2DM). The figure on the left (**A**) shows the AUC for the ROC of the multivariable model with MetS regressed on CVDs. The figure on the right (**B**) shows the area under the AUC of the ROC of the multivariable model of CRD regressed on CVDs.

The AUC values (MetS = 0.8475 vs CRD = 0.7943) for models shown in Table 3 and their corresponding ROC curves are shown in Figure 2. The AUC values suggested that MetS may have better predictive capacity for T2DM than CRD models (Figure 2). For MetS, AIC and BIC values were 1237.012 and 1283.22, respectively. For CRD, the AIC was 1407.87 and BIC was 1454.07, respectively.

## Discussion

In this study, we compared how MetS and CRD were associated with CVDs and T2DM in a representative sample of the general population of Qatar while also controlling for important sociodemographic factors. Notably, both syndromes exhibited significant associations with both outcomes, with MetS potentially showing stronger and more predictive associations than CRD for both of these outcomes.

In alignment with the literature, MetS also showed a strong association with CVDs in our study. This is not surprising given that different components of MetS are also part of the pathophysiological process leading to CVDs.^22^ Evidence suggests that impaired glucose regulation,^23^ obesity,^24^ insulin resistance,^25^ dyslipidemia,^26^,^27^ and hypertension^28^ were contributing risk factors for CVDs. Moreover, this finding can also be explained through the mechanism of chronic inflammation markers. Previous research indicated that MetS was associated with markers of mild and chronic inflammation (eg, high levels of fibrinogen, C-reactive protein, and Erythrocyte Sedimentation Rate) typically associated with insulin resistance.^22^ Normally, insulin enhances albumin synthesis and reduces fibrinogen synthesis in the liver, contrasting with the acute-phase response. However, decreased insulin sensitivity, as seen in MetS, may potentially disrupt this balance, leading to increased production of acute-phase proteins like fibrinogen and C-reactive protein, previously shown to be potential predictors of CVDs.^29^

In our study, CRD was associated with double the odds of CVDs in our sample of Qatar’s general population emphasizing the importance of circadian disruptions in influencing cardiovascular outcomes. This is consistent with findings from two previous cross-sectional studies reporting that CRD may also be an important predictor for CVDs.^15^,^16^ Previous studies also supported that circadian rhythms play an important role in regulating multiple physiological processes including blood pressure, endothelial function and lipid metabolism, all of which are key determinants of the risk of CVDs.^30^,^31^ Adverse cardiovascular consequences of circadian misalignment were also reported in individuals with irregular sleep patterns or shift work schedules.^31^,^32^

In addition to individuals who have shift work schedules or irregular sleeping habits, vitamin D deficiency was also reported to be associated with an increased risk of developing CRD. A recently conducted study reported a two-fold increase in the odds of CRD as a result of vitamin D deficiency.^33^ Given the high prevalence of vitamin D deficiency in Qatar,^34^ vitamin D may also be an important contributor to the underlying mechanism of circadian rhythm disruptions and cardiometabolic outcomes in Qatar and the region.

However, there are some conflicting findings concerning the relationship between CRD and CVD risk suggesting that circadian disruptions may have variable effects depending on population characteristics and specific conditions.^30^,^31^,^35^ In our study, MetS emerged as a marginally superior predictor of CVDs compared to CRD. This is in contrast to findings from prior investigations involving American and Chinese populations, which concluded that CRD may have higher predictive power for CVDs with ORs of 2.92 (95% CI: 2.21–3.86) and 2.83 (95% CI: 2.33–3.43), respectively. We suspect that the superiority in AUCs for MetS in our findings compared to these two previous studies may arise from differences in how CRD was assessed. Specifically, our data for the main components of CRD and MetS were self-reported and based on questionnaires, whereas objective measurements of waist circumference and serum levels of lipid parameters were carried out in these other studies.

Even though both MetS and CRD were associated with T2DM status in Qatar, our findings suggested that MetS maybe potential better predictor of T2DM than CRD in the general population. However, future prospective studies may better disentangle the predictive performance of the two syndromes in relation to these outcomes. Some researchers claim that CRD includes key etiological components, such as sleep disturbance and depression that explain the clustering of cardiometabolic risk factors, illnesses, and associated comorbidities, while MetS does not fully address these factors. For example, Hidese et al explored the association between depression and MetS, their findings emphasized that depression, a component of CRD, can significantly influence cardiometabolic outcomes, highlighting the intricate interplay between psychiatric factors and metabolic health.^36^ As more evidence emerges linking these cardiometabolic risk factors and comorbidities to circadian rhythm disorders, it seems that most cluster components share the same etiology highlighting the important role of the body’s circadian cycle. Thus, it is reasonable to propose that circadian disruption may be driving this commonly seen cluster of risk factors and disorders, including T2DM.^17^ In contrast, MetS directly measures physiological changes that impact conditions such as T2DM, circadian disturbances may have a cumulative rather than an immediate effect on health, which over time could contribute to the development of multiple chronic conditions including T2DM.^37^

Our study had many limitations. Firstly, given the cross-sectional study design, causal relationships between MetS and CRD with both T2DM and CVDs cannot be established. Secondly, self-reported data might have introduced random and systematic errors (recall bias and other misclassification bias). However, data collected based on self-report offer a cost-effective way of measuring and tracking the studied phenomena at the population level. Thirdly, the lack of data on one of CRD components,^17^ non-alcoholic fatty liver disease, could have led to the potential exclusion of patients with CRD from our study. Consequently, it is possible that we were not able to capture the full spectrum of individuals affected by CRD, thus limiting the generalizability of the study’s findings. Moreover, small sample size, especially after adjusting the confounders results in imprecise estimates, which can be mitigated by future studies with larger sample sizes. Finally, the underreporting of depressive symptoms due to stigma^38^ may have contributed to the underestimation of the rates of CRD in our sample.

To the best of our knowledge, this is the first study in Qatar and the Middle East to assess the association between CRD and MetS in relation to CVDs and T2DM. Our study is one of the few studies worldwide that investigated CRD as an independent entity from MetS. Moreover, our study had a large (N= 2332) and representative sample of the general population in Qatar. We used a robust analytical approach with relevant sociodemographic and health-related variables to provide contextual insights into the prevalence and associations of MetS and CRD with important health outcomes for informing targeted public health interventions. Future studies can validate our findings in other populations using objective measures and prospective study design.

## Conclusions

The prevalence of MetS in Qatar’s general population was higher than that of CRD. Both MetS and CRD were positively associated with CVDs and T2DM in a representative sample of Qatar’s population. Compared to Qataris, blue-collar expatriates had lower odds of MetS and CRD as such long-term screening and education programs should focus on Qatari nationals. The present study is exploratory yet paves the way for more studies in the Middle East and other regions of the world to better understand the pathophysiology of CRD, MetS and their associations with CVDs and T2DM for early screening and prevention against its complications.

### What This Study Adds?

- Both MetS and CRD were associated with type 2 diabetes (T2DM) and cardiovascular diseases (CVDs) in Qatar.
- MetS showed potentially stronger associations than CRD for both T2DM and CVDs.

### Implications for Policy and Practice

- Our findings suggest the need for early screening and public education of known risk factors of MetS and CRD.
- Further prospective research is needed for predictive utility of CRD compared with MetS for T2DM and CVDs in different populations including the Middle East.

## Data Sharing Statement

Data are available upon request from the corresponding author.
